# Asp Viper (*Vipera aspis*) Envenomation: Experience of the Marseille Poison Centre from 1996 to 2008

**DOI:** 10.3390/toxins1020100

**Published:** 2009-11-24

**Authors:** Luc de Haro, Mathieu Glaizal, Lucia Tichadou, Ingrid Blanc-Brisset, Maryvonne Hayek-Lanthois

**Affiliations:** Centre Antipoison, hôpital Salvator, 249 boulevard Sainte Marguerite, 13009 Marseille, France; Email: mathieu.glaizal@ap-hm.fr (M.G.); lucia.tichadou@ap-hm.fr (L.T.); ingrid.blanc@ap-hm.fr (I.B.-B.); maryvonne.hayek@ap-hm.fr (M.H.-L.)

**Keywords:** asp viper, snake envenomation, antivenom, neurotoxins, *Vipera* genus

## Abstract

A retrospective case review study of viper envenomations collected by the Marseille’s Poison Centre between 1996 and 2008 was performed. Results: 174 cases were studied (52 grade 1 = G1, 90 G2 and 32 G3). G1 patients received symptomatic treatments (average hospital stay 0.96 day). One hundred and six (106) of the G2/G3 patients were treated with the antivenom Viperfav* (2.1+/-0.9 days in hospital), while 15 of them received symptomatic treatments only (plus one immediate death) (8.1+/-4 days in hospital, 2 of them died). The hospital stay was significantly reduced in the antivenom treated group (p < 0.001), and none of the 106 antivenom treated patients had immediate (anaphylaxis) or delayed (serum sickness) allergic reactions. Conclusion: Viperfav* antivenom was safe and effective for treating asp viper venom-induced toxicity.

## 1. Introduction

Four species of vipers of the *Vipera* genus live in mainland France. Two of these species have not been implicated in causing serious envenomation [1]. Indeed the Basque viper (*Vipera seoanei*) and the meadow viper (*Vipera ursini*) are small snakes that live within a restricted area and are not medically significant [2]. The other two French viper species, *i.e.*, the adder (*Vipera berus*) and the asp viper (*Vipera aspis*) are larger snakes, ranging from 40 to 60 cm in length, and can cause life-threatening envenomation. Since it is adapted to cold regions, *Vipera berus* can be found in Scandinavian countries beyond the Arctic polar circle. The northern half of France and the mountainous regions of central France mark the southernmost extension of the habitat of this species. This snake is common in Sweden and England. *Vipera aspis,* that requires sun and warm temperatures, is found mainly in the regions south of the Loire. It can proliferate in suitable environments. Pattern markings are highly variable, even within the same population. Our reporting of the data concerning *Vipera aspis* bites in Southeastern France over a 13-year period should provide clinicians with useful insights for the management of patients envenomed by native *Vipera* species, including those involving neurological toxicity.

## 2. Composition of Venom and Circumstances Related to Envenomation

The venoms of *Vipera aspis* and *Vipera berus* present similar compositions [1]. Both contain kininogenase (hypotensive bradykinin-releasing enzyme), prothrombin-activating factors, proteases, and hyaluronidases. Neurotoxins of the phospholipase A2 type have been found in the venom of some populations of *Vipera aspis,* but never in the venom of *Vipera berus* (Table 1) [3,4,5,6,7,8,9,10,11,12]. 

The adder and asp viper are not aggressive. The most serious envenomations occur when the snake feels threatened: most severe poisonings happen when the victim either touches the snake unwittingly with his hand or attempts to handle it. Even neonate *Vipera apis* are capable of inflicting medically serious envenomations [1].

**Table 1:** Neurotoxic phospholipases A_2_ from European viper venom.

Vipers	Neurotoxins	Activities	References
Vipera aspis aspis,Nice city area	ammodytoxin A, B, C	Monomeric presynaptic	[3,4,21]
	vaspin	Heterodimeric postsynaptic	[3,4,21]
Vipera aspis zinnikeri, Montpellier city area	PLA_2_-I	Heterodimeric postsynaptic	[5,6]
Vipera ammodytes ammodytes	ammodytoxin A, B, C	Monomeric presynaptic	[7]
	vipoxin	Heterodimeric postsynaptic	[8,9,10]
Vipera ammodytes meridionalis	vipoxin	Heterodimeric postsynaptic	[11,12]
Vipera berus berus	None	None	[4]

## 3. Clinical Features and Grading of Envenomation

Since the beginning of the 1990s a French grading scale for viper envenomations (Table 2) has been validated [13,14], confirmed by several clinical series description [15,16] and is now used in other European countries [1]. Vipers inject venom under pressure and the bite lasts only a few tenths of a second. In some cases there may be only one puncture site. When there are two fang marks, the distance between them may initially be a few millimeters but then increase to over one centimeter as swelling develops. If pain is moderate and limited to the injection sites and if there is no progression of symptoms with time it can be safely concluded that no venom was injected (grade 0) [13,14]. Envenomation from these snakes features immediate pain that can be severe. This pain is likely due to venom components such as proteases, biogenic amines and probably other constituents. Pain is followed within a few minutes by inflammatory edema and sometimes blistering at the bite site (grade 1). Most envenomations stabilize at grade 1 then regress spontaneously after 24 to 72 hours. In mainland France only 15 to 20% of envenomation progress to grade 2. This percentage is variable from one geographic location to another depending on the venomous species. Grade 2 symptoms can either occur rapidly (early grade 2) with the appearance of low blood pressure in the first 30 minutes after injection or be delayed (classic grade 2) for 6 to 16 hours with extensive swelling and sometimes general symptoms (vomiting, abdominal pain, malaise, and laboratory findings showing asymptomatic haemostatic disturbances). Two systemic signs are unfavorable prognostic indicators, *i.e.*, diarrhea and arterial hypotension that does not resolve with fluid resuscitation or provision of colloids [1,15,16].

**Table 2:** Clinical gradation of European Viper envenomations [1,13,14].

Grade	Envenomation	Clinical feature	Treatment	Average venom blood level [13,14]
0	Dry bite	Fang marks, no local signs	Local wound care only	1 ± 0.3 ng/mL
1	Minor	Local swelling, pain, no general symptoms	Symptomatic	5 ± 1.8 ng/mL
2	Moderate	Extensive swelling and/or moderate general symptoms (hypotension, moderate digestive troubles)	Antivenom	32 ± 7 ng/mL
3	Severe	Giant swelling and severe general symptoms (progression of grade 2 symptoms, diarrhoea)	Antivenom	126 ± 50 ng/mL

Grade 3 envenomations are defined as grade 2 symptoms that continue to progress for several hours without specific treatment with development of extensive swelling at the trunk. General symptoms persist at grade 3 with numerous complications that can lead to multiple organ failure (renal insufficiency due to tubulopathy or glomerular nephropathy, hypoxia with variable bleeding due to pulmonary lesions related to edema and sometimes with pleural effusion, multiple episodes of digestive or respiratory bleeding, *etc*.). Laboratory tests may detect abnormalities as early as grade 2 including thrombocytopenia, hyperleukocytosis, and hypofibrinogemia, but severe water-electrolyte imbalance or coagulation disturbances are associated with grade 3.

Studies carried out on blood samples from viper bite victims show that the severity of envenomation is closely linked to the concentration of venom in the blood. Using ELISA to measure venom blood level can predict short-term clinical course (Table 2) [13,14,17]. However this technique is still experimental and is not yet applicable in emergency situations [1].

**Figure 1:**
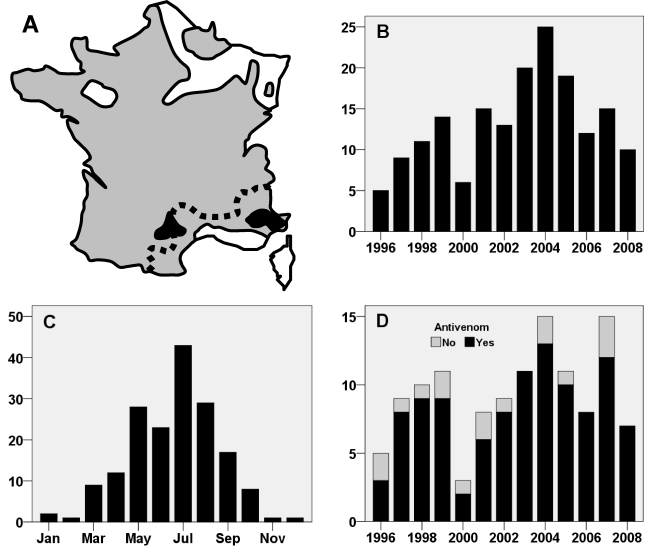
Asp Viper envenomation, experience of the Marseille Poison Centre between 1996 and 2008. In **A**, the geographical distribution of dangerous vipers in mainland France is coloured in grey. The two black areas indicate the places where neurotoxic symptoms are observed. Both of them are included in the Marseille poison centre activity zone (all the French Mediterranean coast, at the south of the dotted line). The chronological distribution of the asp viper envenomation of the case series is presented by year in **B** and by month in **C**. In **D**, annual distribution of grade 2 + 3 envenomations with or without antivenom treatment.

Neurotoxins in the venom of some asp vipers can cause special clinical manifestations with a decrease in local or regional symptoms (although still present after injection of venom) and appearance of general neurologic signs within 4 to 12 hours [18,19]. The most frequent symptom is ptosis but other signs have been reported including ophthalmoplegia, diplopia, dysarthria, paralysis of the orbicularis oris and difficulty in swallowing and focusing (Table 3). More extensive neurological manifestations may be observed with drowsiness, vertigo, dyspnea, and diffuse paresthesia [20]. As shown in Figure 1A, this unusual envenomation pattern has been described only in small zones in southern France [20,21]. The grading system used for typical envenomation (Table 2) is also applicable to viper envenomations involving neurotoxins. In this regard, neurologic manifestations should be considered as general signs and are associated with grade 2 classification [1].

**Table 3:** Clinical feature of 14 neurotoxic viper envenomations observed in the Marseille’s Poison Centre during the 1996-2008 present study. French department codes: **12** = Aveyron, **34** = Hérault, **06 **= Alpes Maritimes and **04** = Alpes de Haute Provence.

French department code	12	34	6	4	Total
**Subspecies**	*Vipera aspis zinnikeri* Montpellier city area		*Vipera aspis aspis* Nice city area		2 subspecies
**n**	3	1	5	5	14
**M/F**	1-Feb	Jan-00	2-Mar	1-Apr	4-Oct
**Age in years min.-max.**	4-62	5	32-68	36-88	4-88
**Grade 1/2/3**	0/3/0	0/1/0	0/4/1	0 /3/2	0/11/3
**Local signs**					
** Local swelling**	2	1	1	2	6 (43%)
** Extensive swelling**	1	0	4	3	8 (57%)
**Neurological symptoms**					
**Ptosis**	3	1	5	5	14 (100%)
**Ophtalmoplegia**	2	1	3	3	9 (64%)
**Vision troubles**	1	0	2	3	6 (43%)
**Dysphagia**	1	1	3	1	6 (43%)
**Dysphonia**	1	1	4	0	6 (43%)
**Paresthesias**	1	0	3	1	5 (25%)
**Drowsiness**	2	1	1	0	4 (29%)
**Lips paralysis **	2	0	1	0	3 (21%)
**Muscle weakness**	1	0	0	0	1 (7%)
**Other general symptoms**	3	0	5	5	13 (93%)
**Antivenom treated patients**	2	1	5	5	13 (100%)

## 4. Treatment Protocol Used for Viper Envenomation in Mainland France

Management of viper envenomation must follow strict rules both at the scene of the snake bite and during transportation to the hospital [1,15].

First aid at the scene immediately after the bite: Immobilize the victim since any type of activity can increase spreading of the venom. Call for assistance as soon as possible. If the bite takes place in a remote area and it is necessary to seek a means of communication, the victim can be carried (e.g., children) but also left at the scene (preferably in the company of another person). The exact location of the bite scene must be specified. While waiting for assistance to arrive, remove tight clothing and jewelry (watches, bracelets, *etc*.) from the bitten extremity to avoid tightening due to swelling and, if possible, disinfect the wound.

What not to do: Do not restrict circulation by applying a tight band or tourniquet. Do not promote spreading by administering drinks that increase heart rate (coffee or tea), performing mutilating acts such as wound incisions, suctioning, or cauterization. Alcoholic beverages and recreational drugs must be prohibited for bitten patients. Antivenom should not be administered without medical supervision. 

What is not useful: Immediate use of heparin or its derivatives is unnecessary. Injection of low-molecular weight heparin may promote spreading of venom. Similarly administration of corticosteroids is not useful. Aspiration devices like pumps cannot extract venom deeply injected in the victim’s tissues.

Management during transportation to the hospital: The presence of any local manifestations indicates grade 1 envenomation and requires hospitalization. Placement of an intravenous line is a necessary precaution to allow immediate vascular filling by macromolecules in case of arterial hypotension. Non-sedating analgesics should be administered to patients in case of severe pain.

Management at the hospital: Prompt clinical assessment (examination and interview) is necessary to establish envenomation grade upon arrival at the hospital (Table 2) [13,14]. Thorough laboratory testing should be carried out including hemogram, hemostasis (platelets, prothrombin rate, INR, fibrinogen, and detection of fibrin degradation products), and kidney functional testing (creatinemia, hematuria, and proteinuria).

If the patient presents grade 0 envenomation, a 4-hour observation period in the emergency room may be proposed. However the need for such surveillance is controversial since absence of pain signifies that no venom has been injected and thus all but rules out the risk of progression from grade 0 to grade 1. 

If the patient presents grade 1 envenomation symptoms, hospitalization is required for at least 24 hours since venom has been injected and it is impossible to predict the outcome. Appropriate supportive treatment should be administered: analgesics, raising and immobilizing the extremity. As infectious complications are rare in Europe antibiotics should be administered only if infection is suspected. In this regard purulent material coming from the site of venom injection is a common early indicator of bacterial growth [1].

If the patient presents grade 2 or 3 envenomation, it is necessary to undertake the only specific treatment with proven effectiveness, *i.e.*, intravenous immunotherapy with antivenom. The only antivenom currently available in France is Viperfav* that contains F(ab’)2 fragments obtained from antibodies produced by immunizing horses with venom collected from *Vipera berus*, *Vipera aspis* and *Vipera ammodytes* of the Balkan area (500LD50, 1000LD50, and 1000LD50 respectively per 4 mL bottle). Viperfav* has been approved for marketing and use only in a hospital setting. Although it appears to be well tolerated (no reports of severe adverse effects), Viperfav* contains heterologuous proteins that require use under surveillance at a medical facility. Approval of Viperfav* was obtained based on studies showing good tolerance and excellent effectiveness as an antidote in envenomated patients [15,16]. Quantities of required antivenom depends not on the victim but rather on the clinical feature and on the quantity and quality of the venom injected that vary depending on the age and health of the snake as well as on the circumstances in which the bite occurs. The pediatric dosage of antivenom is the same as in adults and only the volume of saline used to dilute the antivenom changes. Prompt use of antivenom is recommended for pregnant women in order to neutralize circulating venom that may be toxic for the placenta and fetus [1,15]. It must be emphasized that immunotherapy is the only technique with proven efficacy against grade 2 or 3 envenomation. Preventing the life-threatening effects of envenomation takes priority over the risk of anaphylactic reaction that is manageable most of time in a properly-equipped hospital setting.

Thanks to techniques now available for purification of antivenoms including Viperfav* as well as to current levels of viral security, the intravenous injection route is recommended for immunotherapy. Use of the intramuscular route has been discontinued. In this regard toxicokinetic studies have shown that the intravenous route provides prompt neutralization of antigens not only in the bloodstream but also in tissue [22,23,24,25]. 

The phospholipase A2 neurotoxins present in the venom of some asp vipers in France are similar to the neurotoxins (vipoxin and ammodytoxins) found in *Vipera ammodytes* (Table 1) [3,4,5,6,7,8,9,10,11,12]. Venom from *Vipera ammodytes* is used to immunize horses for production of Viperfav*. Antibodies against neurotoxins from *Vipera ammodytes* have been effective in improving the neurotoxic manifestations observed after snakebites in some areas of France. For this reason use of Viperfav* is recommended for neurotoxic envenomations with no change in dosage: clinical experience showed that one Viperfav* infusion is sufficient to induce a rapid decrease of the neurological symptoms [26].

Infection after snakebite regardless of clinical envenomation grade is a slight but non-negligible risk. However there is no consensus on the need for systematic antibacterial therapy [1,15]. Although the mouth of these serpents is highly septic, infection is uncommon. It is important to monitor the wound site so that treatment can be started at the first sign of infection [1]. Tetanus has never been observed following snakebite in Europe but this type of accident provides an opportunity to check and, if necessary, update tetanus immunization status.

Anticoagulant treatment using heparin or low-molecular weight derivatives has no interest for the management of the direct venom induced toxicity. This treatment is necessary in only two cases. The first indication is to prevent possible venous thrombosis in patients presenting snakebites located on the lower extremity with extensive edema requiring prolonged decubitus. The second indication for anticoagulant treatment involves patients presenting grade 3 envenomation with disseminated intravascular syndrome [1,13].

The role of surgery is limited and currently subject to controversy. In the past fasciotomy was recommended in cases involving extensive edema in order to relieve pressure and avoid peripheral ischemia due to compression. Current experience has demonstrated that immunotherapy using antivenom leads to prompt reduction in edema thus ruling out the risk of compression. Peripheral ischemia was not observed in any patient included in recent series of snakebites attributed to European vipers with or without antivenom immunotherapy. Thus fasciotomy is generally considered as unnecessary for viper envenomation in Europe and used only in rare cases for snakebites by other species (genus *Crotalus*) [1].

## 5. Data Collection

The Poison Control Center of the Marseille Public Hospital System is a clinical toxicologic department providing 24/7 emergency telephone assistance. The Center serves three regions in mainland France, *i.e.*, Provence-Côte d’Azur, Languedoc-Roussillon and Corsica, covering the whole French Mediterranean coastline. There are no venomous vipers in Corsica (Figure 1A). As part of a study to evaluate the therapeutic protocol currently used to treat viper envenomation in France, activity at the Marseille Poison Control Center has been closely monitored since 1996, which corresponds to the date when Viperfav* antivenom became available to Poison Control Centers (availability to all French hospitals since 2000). Data concerning all cases of snakebite recorded in mainland France were collected based on collaboration between the Poison Control Center and the hospital department where patients were treated. The presented case series is limited to the clinical experience of one French Poison Centre and is not linked to a clinical trial (descriptive clinical experience study with no randomized populations or blinded analysis). All clinical and laboratory findings were recorded in a computer database developed specifically for the purpose using Microsoft Access*. Retrospective analysis was performed on data for all cases involving grade 1 envenomation or higher recorded between the beginning of 1996 and end of 2008. Statistical calculation (patients characteristics, performed treatments, evolution of the envenomation, duration of hospitization…) was performed using the SPSS* software package.

## 6. Results

During the 13-year study period, a total of 174 viper envenomations classified as grade 1 or higher were recorded at the Marseille Poison Control Center (Table 4). There were 120 men and 54 women with a mean age of 34 years (range, 2 to 88 years). Breakdown of data according to annual (Figure 1B) and seasonal (Figure 1C showed an unequal chronological distribution of envenomations. Bites almost always occurred in the summertime but a few occurred in the middle of winter (Figure 1C). Analysis of the circumstances surrounding winter envenomations showed that most were inflicted by snakes that were disturbed during hibernation by victims who often reported having placed their hands in a wood stack or in a whole in an old wall.

**Table 4:** Description of the 174 cases of viper envenomations managed by the Marseille Poison Centre between 1996 and 2008 inclusive.

Grade	n	Child/ adult *	Neurotoxic symptoms	Place of medical management				Patients treated with anti-venom	Evolution		
Home	Emerg.	Spe. U	ICU	Rapid recovery	Delayed recovery	Death
**1**	52	Dec-40	0	8%	92%	0	0	0	96%	4%	0
**2**	90	28/62	11 (12%)	0	77%	13%	10%	80 (89%)	89%	11%	0
**3**	32	24-Aug	3 (9%)	3% **	0	3%	94%	26 (81%)	72%	19%	9%
**Total**	174	48/126	14 (8%)	3%	67%	6%	24%	106 (61%)	88%	10%	2%

* There is no significant difference in the grade distribution between adults and child patients with a Chi2 statistical test.

** One patient died in few minutes after the bite by direct intravascular venom injection, and before any medical management.

Emerg. = Emergency room, Spe. U = Specialized Unit, ICU = Intensive Care Unit.

Data breakdown according to severity showed that 52 patients remained at grade 1. Most of these patients (92%) were examined in hospital emergency rooms. They remained a day at the hospital for observation to rule out progression to grade 2 and were released if no change was observed. Appropriate supportive treatment was administered to enhance comfort but antivenom immunotherapy was never used in compliance with the therapeutic protocol. In two cases recovery was delayed due to infectious complications, thus underlining the fact that the toxic and infectious risks are completely unrelated. Patients should be advised of the risk of infection even if envenomation is minimal.

Grade 2 envenomations were most common (n = 90). All grade 2 patients were hospitalized for observation in the emergency room (77%), in specialized units, e.g. pediatric or traumatology departments (13%), or directly in intensive care units if the patient displayed additional risk factors (10%). Although the therapeutic protocol current used in France calls for immunotherapy for grade 2 patients, antivenom was used in only 80 of the 90 cases (Table 4, Figure 1D). It is noteworthy that all patients who received the antidote treatment recovered rapidly with a mean hospitalization period of less than 2 days. In contrast delayed recovery (more than 3 days) was observed in all grade 2 patients who did not receive immunotherapy with a mean hospitalization period of about one week. The difference in mean hospitalization period between grade 2 patients who did and did not receive antivenom immunotherapy was highly significant (Table 5). 

**Table 5:** Duration of envenomed patient hospitalization with or without antivenom treatment.

Grade	Average hospitalization duration (days)				Total (days)
	No antivenom	1 infusion	2 infusions	3 infusions or more	Without/With antivenom
**1**	0.96 ± 0.8	NA	NA	NA	0.96 ± 0.8/NA
**2**	6.8 ± 3.6	1.9 ± 0.85	2.3 ± 0.6	1.75 ± 0.5	6.8 ± 3.6/1.9 ± 0.8 (p = 0.002)
**3**	10.6 ± 4 *	3 ± 0.8	2.4 ± 0.9	2.7 ± 1	10.6 ± 4 */2.6 ± 0.9 (p < 0.02)
**2 + 3**	8.1 ± 4 *	**1.95 ± 0.9**	**2.3 ± 0.7**	**2.4 ± 0.95**	8.1 ± 4 * /2.1 ± 0.9 (p < 0.001)

* The case of immediate death is not included in the calculation of the average hospitalization duration; NA = Not Applicable.

Grade 3 envenomation was less common, with only 32 cases in this series. Patients presenting severe symptoms often associated with clinical and laboratory complications were usually admitted to the intensive care unit. An unusual case of snakebite with direct intravascular injection of venom led to death within a few minutes with no time for management (man, 45 years old, without previous medical history, bitten in the thigh in July 2004). Autopsy demonstrated massive myocardial injury with minimal local manifestations. Since the victim was classified as a home-treatment case in violation of the standard protocol for grade 3 envenomation (Table 4), this case was not taken into account for calculation of mean hospital stay. Most of the other grade 3 envenomations involved patients in whom management was delayed or in whom grade 2 was allowed to progress to grade 3 due to failure to provide antivenom in time. Five of the grade 3 patients that were managed in the hospital did not undergo immunotherapy including two that died despite intensive care management (women, 69 years old, without previous medical history, bitten in June 1996 on the foot and died after 4 days in ICU due to hemorrhagic and pulmonary complications; women, 45 years old, without previous history, bitten in June 2005 on the foot and died after 4 days in ICU due to cardiac and pulmonary complications) and three in whom recovery was delayed. The mean duration of hospitalization in patients who were not treated with specific antivenom therapy was 10.6 days. This duration that is probably negatively skewed by the short stays of the 2 patients who died is still significantly higher than the 3-day mean observed for patients who were treated with antivenom. This finding demonstrates that immunotherapy is useful even for patients in whom management has been delayed or in whom tissue complications have already developed.

Breakdown of envenomation severity according to patient age showed no difference in grade distribution between adults and children less than 15 years (age considered as the best cutoff to distinguish lower body weight and less resistant young cases from standard adult cases). This finding indicates that there is no need to modify the therapeutic protocol for children [1]. 

Multiple Viperfav* perfusions did not reduce the duration of hospitalization of grade 2 and 3 patients (Table 5). In this regard it should be underlined that the initial protocol calling for repeating antivenom infusions every 4 hours until observation of clinical improvement was proposed before marketing of Viperfav* began. Evidence based on subsequent clinical experience of French Poison Centres has shown that a single dose of antivenom contains sufficient antibodies to rule out the need for repeated infusion : unpublished data from the Angers Poison Control Center (Workshop on envenomation by vipers in Europe and their treatment with antivenoms, National Museum of Natural History in Paris, November 10, 2006) showed that venom levels in the blood of patients treated with Viperfav* decrease after the first round of antivenom and never increase thereafter. Our results are in agreement with this finding and thus indicate that multiple infusions are unwarranted. Regarding tolerance to Viperfav*, it is important to note that no immediate (anaphylactic reaction) or delayed (serum sickness) adverse effect was recorded during prolonged follow-up of the 106 patients (Table 4) who underwent a total of 162 Viperfav* infusions.

**Figure 2:**
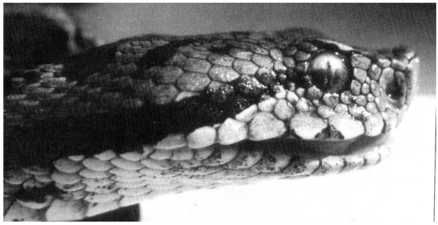
The snake with the typical morphology of *Vipera aspis aspis* and responsible for one of the first neurotoxic envenomation observed in 1992 near the city of Nice (Italian border).

In two circumscribed locations in France envenomation was characterized by neurotoxic manifestations (Figure 1A). In this series a total of 14 patients bitten in these areas presented clinical manifestations different from those observed elsewhere (Table 3). Recent studies show that the neurotoxins in the two populations belonging to different subspecies are different (Table 1 and Table 3, Figure 2) [20,21,27]. 

In our series all patients who presented neurotoxic manifestations responded rapidly to immunotherapy using Viperfav* and recovered within two days. These results confirm that this antivenom containing antibodies against neurotoxins from *Vipera ammodytes* is effective in neutralizing neurotoxins from both populations of « neurotoxic » asp vipers [26]. It is also noteworthy that another supposed neurotoxic snake species lives in the southern France, *i.e.*, the Montpellier snake (*Malpolon monspessulanus*), and that its habitat covers both regions in which the neurotoxic vipers are found in France. However it is unlikely that confusion of two species occurred since the Montpellier snake is larger and more aggressive than the asp viper. In addition rare descriptions of envenomations by *Malpolon* are characterized by slight or minimal local signs even if neurotoxicity is observed immediately [28]. This pattern was never observed after asp viper envenomation in our series.

## 7. Discussion

The asp viper is rightly considered as the most dangerous venomous snake in mainland France. Despite its relatively small size and fairly non- aggressive behavior, this species has been implicated in numerous grade 2 and 3 envenomations. It is important to note that the antivenom now available in France is a well-purified product that is safer than most other antivenoms (no adverse effect observed in our series or registered by the French pharmacovigilance system). The argument that the cost of this product is prohibitive (about 1,000 euros per infusion) is untenable in the light of published data [15,16] and the results of our series showing that use of antivenom significantly decreases the length of hospitalization by several days. Thus the product cost is offset by savings on other health care expenses. Although there is no reason for not using this antidote for grade 2 and 3 envenomation, some clinicians are still reluctant Figure 1) due to past experience with earlier poorly purified antivenoms that are no longer available in France. At the present time failure to administer a dose of Viperfav* to grade 2 or 3 patients should be considered as a serious treatment error. 

Analysis of medical records for this study also revealed that many treatment teams expressed an opposite concern by asking why antivenom immunotherapy was not indicated for grade 1 envenomation. Indeed our data describing follow-up in the days after release from the hospital was that all patients who underwent prompt immunotherapy using Viperfav* for grade 2 envenomation were fully recovered upon returning home whereas most grade 1 patients presented persistent minor symptoms, *i.e.*, edema, pain (sometimes causing disability) and functional disturbances, for several days despite appropriate symptomatic treatment. Despite this discrepancy, French authorities maintain the position that improvement in patient comfort does not warrant the use of costly heterologous proteins with a potential allergic risk [15,16].

This study also confirms the presence of viper populations with neurotoxic venom in southern France as initially reported in the early 1990s [18,19,21]. This finding underscores the evolving nature of venoms. Venoms are subject to multiple environmental factors and thus to constant evolutionary pressure. This fact must be taken into account by medical practitioners that must be able to adapt management protocols quickly and to admit that accepted clinical and therapeutic standards can become obsolete. With regard to neurotoxic venom it should be noted that neurotoxic asp vipers have also been observed in Italy [29]. 
